# Integration of Multisensor Hybrid Reasoners to Support Personal Autonomy in the Smart Home

**DOI:** 10.3390/s140917313

**Published:** 2014-09-17

**Authors:** Miguel Ángel Valero, José Bravo, Juan Manuel García Chamizo, Diego López-de-Ipiña

**Affiliations:** 1 Departamento de Ingeniería y Arquitecturas Telemáticas, Universidad Politécnica de Madrid, Carretera de Valencia km. 7, Madrid 28031, Spain; 2 MAmI Research Lab, Universidad de Castilla La Mancha, Ciudad Real 13071, Spain; E-Mail: Jose.Bravo@uclm.es; 3 Universidad de Alicante, Alicante 03690, Spain; E-Mail: juanma@dtic.ua.es; 4 Deusto Institute of Technology, DeustoTech, University of Deusto, Avda. Universidades 24, Bilbao 48007, Spain; E-Mail: dipina@deusto.es

**Keywords:** Ambient Assisted Living, intelligent systems, sensors networks, smart home

## Abstract

The deployment of the Ambient Intelligence (AmI) paradigm requires designing and integrating user-centered smart environments to assist people in their daily life activities. This research paper details an integration and validation of multiple heterogeneous sensors with hybrid reasoners that support decision making in order to monitor personal and environmental data at a smart home in a private way. The results innovate on knowledge-based platforms, distributed sensors, connected objects, accessibility and authentication methods to promote independent living for elderly people. TALISMAN+, the AmI framework deployed, integrates four subsystems in the smart home: (i) a mobile biomedical telemonitoring platform to provide elderly patients with continuous disease management; (ii) an integration middleware that allows context capture from heterogeneous sensors to program environment's reaction; (iii) a vision system for intelligent monitoring of daily activities in the home; and (iv) an ontologies-based integrated reasoning platform to trigger local actions and manage private information in the smart home. The framework was integrated in two real running environments, the UPM Accessible Digital Home and MetalTIC house, and successfully validated by five experts in home care, elderly people and personal autonomy.

## 1. Introduction

The Convention on the Rights of Persons with Disabilities, put forth by the United Nations in 2006, points out the need “to undertake or promote research and development of, and to promote the availability and use of new technologies, including information and communications technologies, mobility aids, devices and assistive technologies, suitable for persons with disabilities” [1]. The research detailed in this paper aligns with this approach by supporting personal autonomy of users during daily life activities carried out in the home environment. The Ambient Intelligence (AmI) paradigm aims to take advantage of Information and Communication Technologies (ICT), devices and appliances that widely surround people to make life “easier”. The interoperation at home of these available mechanic, electronic, ICTs sets the basis to provide elderly population and/or people with disabilities or chronic diseases with accessible services to promote independent living. The aging-in-place concept, cited by Cheek in 2005, looks to accessible facilities in the Smart Home (SH) to support “emergency care, fall prevention & detection, reminder systems and assistance for those with cognitive impairments” [2]. This idea, described by Williams *et al.* in 1998, envisaged a future smart home for “the provision of artificial intelligence-AI-based information processing and the management of decision-making structures required” [3]. Market trends have fostered affordable wired and wireless sensors in the residential environment house to monitor presence, motion, fire, flood or gas. Furthermore, biomedical data devices to measure pulse, temperature, glucose or blood pressure in a reliable way are connectable. However, the easy to use and knowledge-based person centered interaction with these devices is still not solved, especially to support the aforementioned SH facilities. TALISMAN+, the integrated system that is described in details in this paper, aims to innovate in this research field by demonstrating a hybrid solution that includes the following subsystems: (i) a mobile biomedical telemonitoring platform; (ii) an integration middleware to manage heterogeneous environmental and personal data sensor networks; (iii) a vision system for intelligent monitoring of daily activities and (iv) accessible security mechanisms to guarantee privacy and confidentiality of homecare telematic services.

ICTs can provide people with facilities related to communication, stimulation and environmental control. Laiseca *et al.* put into practice the Ambient Assisted Living (AAL) concept to assist elderly people with cognitive disabilities, by using memory games that facilitate information to caregivers and relatives [4]. Data driven information provided by users or the utilization of sensor-based recognition helps support activity recognition capabilities. The comparison made by Chen *et al.* concludes that Knowledge-Driven models need to handle uncertainty and time in order to distinguish intent or goal recognition [5]. The validation of ontologies systems to support mobile active health was reported by Docksteader *et al.*, in a Mobile Ontology-based Reasoning and Feedback system that monitors SpO_2_, using Sample Semantic Web Rule Language (SWRL), to communicate them via SMS and HTTP [6]. The health care domain has also experimented with the use of cameras in private spaces in AmI, AAL and active aging fields. The review from Cardinaux *et al.* about the pros and contras, details remarkable considerations about user's acceptance, reliability and privacy, of video-based technology for AAL [7].

TALISMAN+ provides facilities that can be critical for the security of vulnerable people with disabilities or chronic diseases. Therefore, as Islam *et al.* states for SH, privacy of monitored individuals should be guaranteed at the same time their identities are checked [8]. Since sensors and devices could become an intrusive element at home, privacy and authentication issues were an essential part of this research to reinforce the trust of users to promote their personal autonomy. System results, designed and deployed according to the needs of two users domains—vulnerable people with Parkinson disease and persons with mobility restrictions—were tested in two real running environments in order to support personal autonomy at the smart home: the UPM Accessible Digital Home and the MetalTIC house.

The structure of the paper is as follows. Section 1 (Introduction) provides a brief review of the AmI challenges and previous works at the Smart Home; Section 2 (User & Services Oriented Architecture) describes the system framework by following a user-centered approach; Section 3 (Results and Discussion) includes the deployment, validation results and discussion for the integrated TALISMAN+ subsystems; and Section 4 (Conclusions) provides an extract of the learnings and feedback from this research.

## 2. User & Services Oriented Architecture

The design and experimental stages of the resulting research followed a user-oriented approach in order to build the system architecture according to the 4 + 1 views of ANSI/IEEE 1471–2000. The steps suggested by this standard helped identify and face the global expectations of a person with cognitive or mobility restrictions who becomes the main stakeholder addressed by each of the five deployed subsystems. This approach led us to define an integrated solution at the SH by providing context-aware reactions triggered by detected events, user profiles and hybrid reasoning procedures. The main concerns addressed in the integrated system deal with verifiable decision support approaches, wireless data acquisition, private monitoring, accessible security, reliability and interoperability. Security view followed a user-centered approach so that an aging person may feel trust about his or her interaction with the system [8]. Therefore, user requirements analysis led us to design, develop and validate a graphical user interface so that all the stakeholders may simulate, check and effectively verify the suggestions provided by the system at the SH. These stakeholders vary from the elderly or disabled persons, informal caregivers, relatives, and professionals at geriatric, primary, therapy, social or nursing care.

The specification of end-user-driven data acquirement at the SH is a key issue to define context-aware interoperable facilities for active living [9]. Knowledge acquired from users' entities, 10 patients from Madrid Parkinson's disease association and 10 users from the Association of People with Spinal Cord Injury and Physical Disabilities (ASPAYM) enriched this stage through replicable user needs.

**Figure 1.**
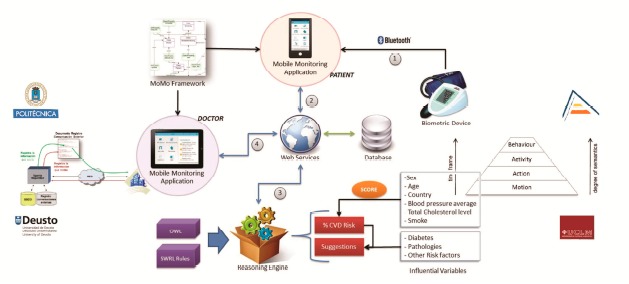
TALISMAN+ Integration of heterogeneous sensors and reasoning model.

Figure 1 shows the global architecture of TALISMAN+ that helps understand the user oriented services view by connecting the functionality of the following integrated subsystems: (1) mobile and connected sensors and devices monitor biomedical information from chronic patients or people with disabilities; (2) a vision system provides private information about behaviors, activities, actions and motion at the smart home; (3) the Ontology Web Language (OWL) reasoning engine acquires data via web services, validates user profiles (sex, age, biomedical data, risk, influential variables,…), and sends this information to the hybrid local reasoner that manages uncertainty and triggers context-aware actions at the SH to promote personal autonomy; and (4) a secure telematics wrapper that provides local reasoning from monitored data, authentication, confidentiality and integrity of managed information.

## 3. Results and Discussion

This section details the integration complexity of the multisensor hybrid reasoners by specifying the results from developed and deployed subsystems in a sequential order. The modular structure, data managed, local information system and interoperation facilities of each subsystem is described.

### 3.1. Mobile Monitoring (MoMo) Platform

The Mobile Monitoring platform (MoMo) allows elderly patients to enjoy continuous disease(s) control and direct communication with their doctor or health care agent. This platform enables patient mobile-tele monitoring by using handheld medical monitoring devices (glucometers, blood pressure meters) to send data to a mobile phone via wireless technologies such as WiFi, NFC or Bluetooth [10]. An ontological model was created in order to catalogue the elements and to provide the TALISMAN+ local and remote reasoners with ad hoc feedback for suggested tasks in the smart home.

Patient monitoring represents one of the key elements in the progress and control of illness [11]. This monitoring provides the doctor with continuous data about relevant parameters (vital signs, pulse glucose,…) so that the doctor can accordingly readjust the initial treatment(s) and prescriptions. The mobile phone was the selected technology as it is fairly frequently used by elderly people and it can support daily activities regarding communication and information management.

**Figure 2.**
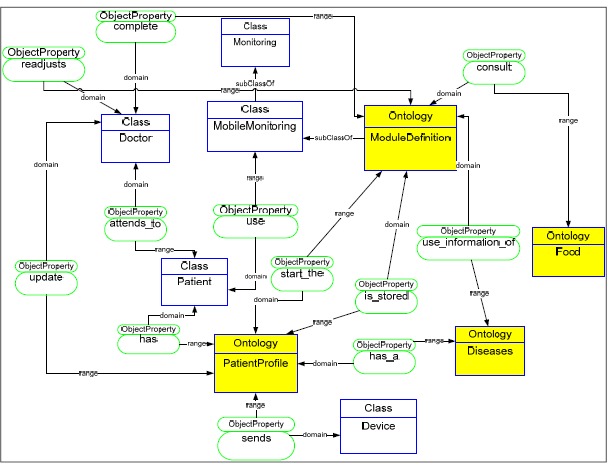
General Ontologies Diagram for mobile monitoring (MoMOntology).

A group of ontologies called “MoMOntology” represents the ontologies of the mobile monitoring process and allows modeling the data which is collected from different biomedical devices (see Figure 2) [12]. The MoMOntology functions facilitate the data storage, infer new information from existing information and guide the generation of applications and their customization to each patient according to that ontological model.

An analytical engine combines Fuzzy Logic and probabilistic reasoning, allowing the management of patient records based on analysis of past situations to predict future difficulties like vital sign changes. The platform takes advantage of mobile phones and biomedical devices to facilitate customized patient monitoring and data recording in a central server that is used by the TALISMAN+ integrated architecture. Security wrappers are in charge of authentication, data cyphering and integration management according to privacy restrictions and legislation.

**Figure 3.**
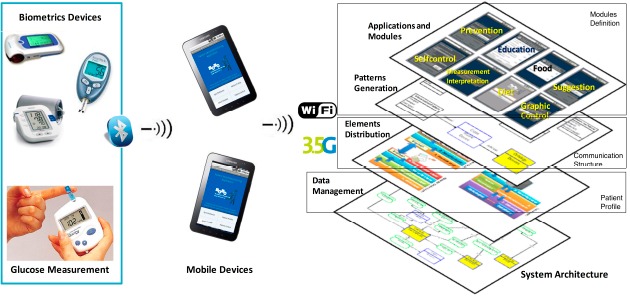
MoMo platform and relationship between data and device entities.

Figure 3 shows the developed platform. Biomedical monitoring devices interconnect with mobile devices through Bluetooth. These medical devices link to a mobile device to process sensor data and to manage applications using redundant connectivity via 3G and WiFi data networks. This generated data is transmitted to a central database at the medical server for evaluation by an inference engine; providing continuous patient monitoring, individual profiles, and a patient module for self-control. The platform supports the communication between patient and doctor.

The platform has three important elements: Patient Profile, Modules Definition, and Communication Structure [13]. The data generated defines the patient profile. The patient profiles have each patient's characteristics. The platform defines a common structure for the patient data (ID, Name, Address, Phone Number, and others). The development of applications and modules specify the modules definition. The pattern definition and the communication structure are defined by elements distribution like patient profiles, modules, and data storage.

The platform allows determining the whole set of modules, via pattern definitions that establish relations between each module and the individual patient profile. The modules define patterns to embed information in the individual profile of each patient. The modules definition, the relations among them, and the individual profile, make possible the generation of modules for the doctor and patient in a mobile phone.

The communication structure takes into account a communication protocol for the measuring devices for each kind of condition, the storage tendencies and the doctor and patient modules. From a physical point of view, a device sends to the system via Bluetooth the data obtained from its sensor. Next, the presented intermediate layer formalizes the received data, retrieving them in a XML document to be deployed by the platform module, depending on its needs. The XML document provides the initial datasheet formalization of the specific device. This common formalization from the original datasheet or specifications enables to communicate with every sensor device in the environment. A mobile development cycle is defined for the development of the platform structure. The proposed development cycle allows us to obtain different functional prototypes that define each element or module that comprise the final platform.

All these elements are embedded into the mobile device; providing the monitoring of all patients' records, organized by patient profile, history of the clinical measures of the patient, and specifications of previously recorded disease(s). The communication module and the platform are developed in Android Operating System with remote connectivity to MySQL database system. The database and all the specifications of the platform are located on the main server; the doctor and the patient have access to these data anytime they use the mobile platform. The patient has a screen with a menu that allows obtaining access to all functions of the application. He/she can update the information in the profile by changing the corresponding parameters. Each measure collects data that is associated with a specific patient id, which allows its updating to the server. Thus, the doctor can interpret the measurements of each patient without confusing them with others. The measurement reading can be manual, so that the patient enters the value through the keyboard, and automatic where the measure is obtained directly from the biomedical device via Bluetooth technology. The patient can select the type of disease that he/she wants to graph, from data captured from the biometric device. The platform can display graphs of different diseases. Our first assessments were considering measures of diabetes, blood pressure, and temperature. These graphics or tables of measures depend on the type of disease(s) of the patient (in some cases there may be more than one) and the changes obtained during a time period. The patient can see his/her latest measurement obtained either through a chart showing the extent and period of time that it was taken, or through a table that stores information about the day and time, as a numerical value, by representing the range of the measure (low, normal, high).

Making easier the mobility of the patient must be an aspect to take into account during the development of applications for monitoring of chronic diseases. This platform has tried to facilitate the independence of people and improve their quality of life. Therefore, the application must allow the mobility of the patient while providing continuous monitoring. Data and notifications of risk situations must be communicated to the medical staff. The definition of the profile of the patient with the information of the disease allows medical practitioners take the most appropriate decisions to facilitate its day-to-day activities. The results obtained in the evaluation with users allow us to know the effectiveness and robustness that offers the application, regarding data processing and control message generation.

We present an evaluation of the functionality of the developed platform. Patients with diabetes and blood pressure, who need to follow-up their illness, have evaluated these domains. We will assess aspects of content, design and usefulness of platform. To evaluate the criteria of content, they responded to questions related to the organization of the content, the presence of help during the use of the platform, ease of interpretation, ease of identification of the elements and the value degree of the sent content. For the design criteria, they responded to questions about the distribution of visual elements, displays, interpretation and identification of menu specifications. For the usefulness criteria, they responded to questions about the usefulness degree of the generated recommendations, prevention and education messages, and satisfaction degree of the information shown.

**Figure 4.**
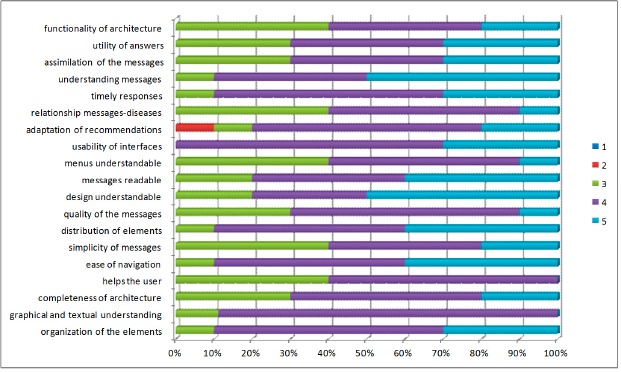
Quality aspects by the end-user assessment.

*Criteria 1. Content*: most participants considered the content of the application as good, while ease of navigation scored between 4 and 5, with high understanding of the elements of the application, and clarity in the information shown (Figure 4). The content is one of the aspects that changes over time in such a way that better and more detailed information is offered to the patient according to the disease that he/she has.
*Criteria 2. Design*: the design criteria was the feature more positively evaluated by the participants. Scores between 4 and 5 according to the total number of participants have been obtained. Most patients responded that the platform was easy to use and the screens could be understood easily. This is because it has a careful design to familiarize patients with the application. The everyday use of the application will make the patient feel better and more secure in the follow-up of the disease.
*Criteria 3. Utility*: in general, the results of the evaluation carried out in this first approach, have been quite satisfactory. The aspects that have low scores have been those related to the utility aspect; this may be due to the fact patients used a diabetes book to make annotations, and sometimes they do not make annotations in the system, so do not perceive the difference when using the evaluated application. Another reason may be that difficulty that some patients have for using this type of technology, where age or inexperience in the use of mobile devices, make them feel some rejection to new technologies because of they consider it takes time to learn. These results helped us to detect possible improvements in such a way that the user should be offered a more robust and friendly architecture.

### 3.2. Hybrid Cooperative Reasoner

**Figure 5.**
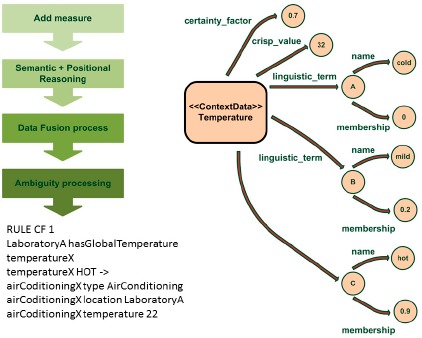
Hybrid reasoner uncertainty management and AMBI2ONT Ontology.

TALISMAN+ framework required development of TALIS+Engine middleware to facilitate context capture from any device, including embedded ones, to program the environment's reactions. The TALIS+Engine subsystem includes the AMBI2ONT ontology [14] created to model ambiguity in its two facets, uncertainty and vagueness, together with sensor fusion and a reasoning inference engine. For uncertainty, the certainty factor (CF) of contextual data is modeled. Vagueness consideration allows modeling unclearly defined situations like cold room or noisy room where different users have different perceptions. Such an ontology models places, and things in those places such as devices or people, capabilities and linguistic terms. Figure 5 shows a ContextData individual with a sensor value associated with a certainty degree about the credibility of such a measure and a set of linguistic terms where each term is associated with a membership function. Thus, the temperature sensor in a given room can be considered mainly hot. Once the measures are modeled considering the uncertainty and the vagueness of the data, a semantic inference process is applied, so that implicit data is derived from explicit data.

For example, the location associated with each measure is determined, knowing the location of the temperature sensor. Subsequently, a data fusion process is applied which aggregates measures of the same type (e.g., temperature sensor) within the same container (e.g., room). The reasoning supports two different strategies: tourney (the best) and combination, where depending on the scheme applied the best, worst or average measures can be considered. Thus, it combines the temperature values of all sensors within a room. Finally, the behavior rules defined for an extension of the JFuzzyLogic fuzzy logic engine are executed. Such an engine was modified to incorporate into it treatment of both uncertain data and rules. Figure 4 shows, on the bottom left hand side, an example of the now supported syntax, which extends JFuzzyLogic engine's Fuzzy control language (FCL), where both uncertainty (CF 1) and vagueness (HOT) are considered.

**Figure 6.**
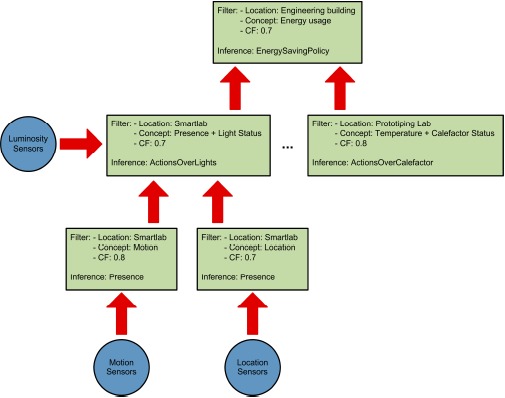
Reasoning hierarchy depicting the sensors (circles) and reasoning engines (squares) that take part in the inference process.

Apart from the need to support context ambiguity management, next generation reasoners for Smart Environments have to support reasoning distribution. As the number of triples in the ontology increases, the inference time for environment actions becomes unsustainable. In order to be able to deploy these systems in real environments, they must be able to react to context changes in a reasonable time for the reaction to be considered appropriate. To address this, we have decided [15] to split the inference problem among different reasoning engines or context consumers according to the interests stated by each of them. The inference is no longer performed by a central reasoning engine, but rather divided into a peer-to-peer network of context producers and context consumers. Dividing the inference problem into smaller sub-units makes it more computationally affordable. Context consumers only receive the information they are interested in and do not have to process non-relevant data. Another benefit is that the different parts of the reasoning process can take place in parallel, reducing the global time taken to resolve the inference problem. The system relies on splitting the reasoning process (see Figure 6) into smaller inference units in order to achieve the following results:

(1). Attain the temporal decoupling of the different inference units. This will allow doing the inference concurrently in various reasoning engines. The parallelization of the inference process reduces the time required to reach certain conclusions.
(2). Attain the spatial decoupling of the inference process. This will increase the general robustness of the system making it more fault-tolerant. Problems in one reasoning engine will not stop all the inference, as opposed to a centralized approach.
(3). Reduce the number of triples and rules that each reasoning engine has to manage. This allows the use of more computationally constrained devices to carry out the inference process.
(4). Compartmentalize the information according to the different interests of the reasoning engines. This will reduce the amount of data that is shared over the network.
(5). Allow the dynamic modification of the created organization. Devices in modern smart environments can change their location frequently (e.g., mobile phones, mote). The created hierarchy must change to adapt itself to these modifications.

In order to decide how the reasoning should be divided among the reasoning engines, three factors are taken into account:

(1). *The ontological concepts that will be used by the reasoner*. These concepts are organized in a taxonomy depicting the class relations of the ontology. The AMBI2ONT ontology described earlier is used. When trying to find an appropriate context provider, the context consumer can search for specific concepts (in our example “Luminosity”) or broader ones (as “Electrical Consumption” that encompass “LightStatus”, “AirConditioning” and “ApplianceStatus”).
(2). *The location where the measures originate from*. As with the ontological concepts, we have a taxonomy of the locations extracted from our ontology. This taxonomy models the “contains” relations between the different rooms, floors and buildings (see Figure 6). The context consumer can search for a specific location (the Smartlab laboratory in our example) or for a set of related locations (for example all the rooms in the first floor of the engineering building).
(3). *The certainty factor* (*CF*) *associated to each ontological concept*. As discussed earlier when modeling real environments, taking certainty for granted is usually a luxury that a context management framework cannot afford. Reality, and thus the context, are ambiguous. Sensors and devices are not perfect and their measures carry a degree of uncertainty, thus several thermometers in the same room can provide conflicting temperature measurements. For this reason, the context consumer can specify a minimum CF in its searches.

To create this hierarchy we have used an agent-based architecture with two types: context providers and consumers. Context Providers are agents representing those elements in the architecture that can provide any context data. Context Consumers are agents representing those elements that need to consume context data. The architecture was implemented by using the Java Agent Development Framework (JADE). This is a software framework implemented in Java that simplifies the implementation of multi-agent systems through a middleware that comply with the Foundation for Intelligent Physical Agents (FIPA, http://www.fipa.org/) agent specifications.

### 3.3. Vision System for Intelligent Monitoring

Using video cameras in private spaces like the SH could suppose the birth of novel AAL applications, particularly in the areas of health and ageing in place [16]. Smart cameras [17] can be used to analyze video streams targeting some incidents addressed in TALISMAN+ like people falling, shower accidents, thief intrusions, and so on [18,19]. Nevertheless, in order to use these kinds of cameras in private spaces the preservation of the privacy of the inhabitants must be guaranteed. Video cameras are low-cost devices that can extract massive amounts of information from the environment using only computer vision technologies. Besides, this sensing technique is the only one that does not put require additional energy to obtain information. Human action recognition constitutes the first level in which a semantic understanding of human behavior can be obtained. Once motion is detected in a scene, commonly a region of interest is obtained using background subtraction or human detection techniques. Then, generated global representations take into account the body's shape and motion over time. In this sense, using human silhouettes as input, the motion history and energy images (MHI, MEI) in which the age and the spatial location of pixel-wise motion are encoded, respectively.

Privacy is protected when there is no association or mapping between sensitive information and a person's identity. Moreover, the information considered as sensitive depends on each person. To protect an individual's privacy, we can: (i) process the visual information in an anonymous way or using blind vision; or (ii) redact videos and images to protect private information. However, current research focuses in redaction methods mixed with data hiding schemes [20]. Image and video modification or redaction methods rely on computer vision algorithms to determine privacy sensitive regions of the image (faces, full bodies, car plates,…) that must be modified. Sometimes, reduction methods are jointly used with data hiding schemes in order to embed original information inside the modified version. There are many ways of image modification, such as private information removal leaving a black hole, use of blurring, pixelating and others commonly used image filters; or use of more robust methods like image encryption and face de-identification.

Initially, the type of human behaviors to be considered as actions are aimed to be recognized during the execution of the subsystem. For this purpose, the state-of-the-art work was reviewed to provide a common definition for the different levels of human behavior. Two scales have been taken into account: (1) the amount of time during which the recognition needs to be performed; and (2) the degree of semantics that is involved in the comprehension of the behavior. In this sense, we define actions as human motion over a time frame ranging from seconds to minutes in which simple human primitives such as standing, sitting, walking and falling can be recognized. The developed approach uses RBG images, and a silhouette-based multi-view human action recognition method. Relying on traditional RGB cameras, color images are processed by means of background subtraction techniques in order to obtain human silhouettes, which serve as input to our method. Not the whole silhouette data is used, but only the contour points that encode the shape of the person and therefore its pose. We have proposed a low-dimensional feature in which a radial scheme is employed in order to spatially align the contour points.

**Figure 7.**
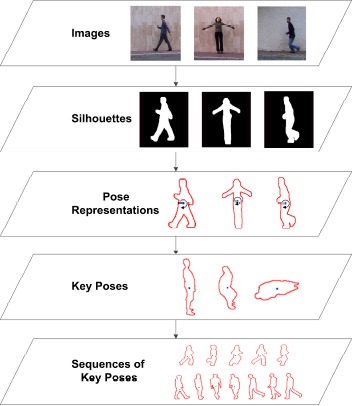
Overview of the proposed human action recognition method.

For each radial bin, a summary value is generated from the statistical range of the distance between the contour points and the centroid of the silhouette. This results in a very low-dimensional feature that filters noise related to inaccurate segmentations. Multiple cameras focusing the same field of view are also considered applying feature fusion techniques. Using the bag-of-key-poses, the most representative poses for each action class, the so-called key poses, are learned (see Figure 7). These are obtained with the K-means clustering algorithm using the cluster centers as representative instances. In contrast to traditional bag-of-words models, we do not perform recognition comparing the frequency of appearance of key poses, but learn the transition between key poses building sequences of key poses.

These are learned by substituting each pose with its nearest neighbor key pose, and therefore, changing the domain of the acquired data to the bag of key poses and filtering noise and sample-specific differences. In order to recognize a new sequence, the same steps are taken until an equivalent sequence of key poses is obtained. Recognition is then performed by means of sequence alignment using dynamic time warping (DTW). Experimentation has been performed on several publicly available datasets as Weizmann, MuHAVi and INRIA XMAS.

**Figure 8.**
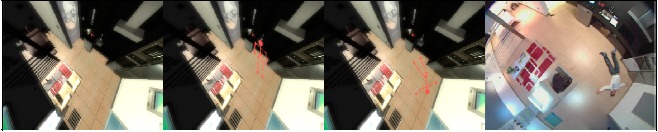
Real images at MetalTIC of integrated privacy levels: scenario with no people, including position, adding gesture to position and the original image.

In order to preserve privacy, we propose a level-based privacy protection scheme, where each level defines its own display model that determines how the captured scene is represented to the observer. Display models are responsible of rendering diminished representations of persons and objects appearing in the scene, and such task may involve removing from the scene all other people or activity that is not of interest for the observer. Used display models are pre-selected according to the exhibition degree of the individual's sensible information and identity in the processed output. Thereby, using distinct display models we can provide several protection levels, from completely protected to unprotected, and observers with camera access can only view the information they are allowed to. We have addressed the privacy of persons subject to monitoring through four different display formats of visual information: from the omission of the people, through virtual representations of the person in the scene, with or without the actual posture, to the accurate representation of the scene including the person. Finally, it was developed a software prototype that implements an early version of this level-based privacy protection scheme. Figure 8 shows the result where, depending on its posture, the person is removed from the processed image and replaced by their skeleton or just blurred.

### 3.4. Integration with Local Logic Description Reasoner and Security Subsystem

The distributed system deployed in this research should comply with the critical security needs that elderly and disabled people may have in the home environment. Local reasoning should validate users' identities and ensure privacy when they are monitored. Sensors and devices used for these services are often perceived as an intrusive element at home. Therefore, privacy and authentication is an essential part of TALISMAN+ not just to protect communications, but also to reinforce users' trust in the use of the services for active aging and promotion of personal autonomy.

**Figure 9.**
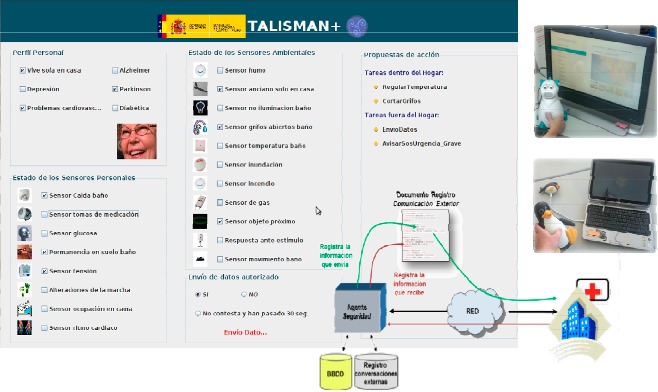
TALISMAN+ security supporting local reasoning engine.

Bearing in mind this user-centered approach, Figure 9 shows the validated interface where the SH logs all interactions of environmental sensors and private monitoring systems that are managing data with the database as well as the data exchanged between the SH and authorized care centers. In this way, users with cognitive or physical disabilities gain access at any time to the information obtained from the logs, as the system enables tangible and understandable interaction when coupling sensors and actuators into actions. These actions allow users to represent the capabilities of the SH where they perform their daily tasks, regardless of the complexity of the underlying ubiquitous system. A security agent is in charge of sending, securing and logging the outward interactions. The agent receives data from SH and establishes a secure SSL channel between home and telecare entities to provide two-way authentication, non-repudiation, confidentiality and integrity services for the exchange.

For this purpose, an XML register document was specified to record all the sequence of interactions that take place between the server and the SH as a result of the execution of the service. After ensuring the security of communications between the house with the outside, there is still an important point to solve referred to the way that people at home are identified. Mutual authentication in SSL required users must be in possession of the corresponding X.509 certificate to operate the system.

**Figure 10.**
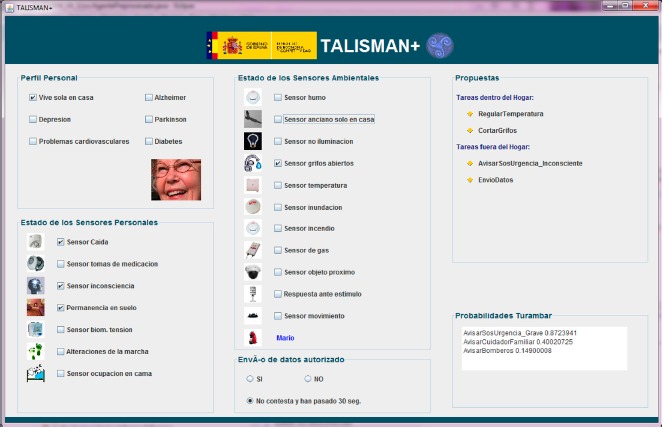
Representation of data for local and hybrid TALISMAN+ reasoners in the Smart Home.

The quality of the behavior of the integrated local hybrid reasoner was evaluated by measuring the outputs from a set of heterogeneous data inputs that come from the combination of 7 personal sensors and 11 ambient sensors deployed for the global system. Figure 10 shows the TALISMAN+ status and system results for the use case of a person with mobility restrictions who lives alone at the SH and fell down meanwhile she was in the bathroom. At the upper left of the Graphical Interface, the User Profile (Perfil Personal) indicates that the person lives alone at home (Vive sola en casa) and has no Alzheimer's, Depression, Parkinson's, Cardiac Disease or Diabetes diagnosis. If so, the hybrid reasoning rules would change the behavior of the system. Below the User Profile box, the system shows the Status of the seven Personal and Biomedical sensors (see Estado de los Sensores Personales in Figure 10) where the Fall sensor (Sensor Caida), the No answer sensor (Sensor inconsciencia) and the Stay on Floor sensor (Permanencia en suelo) are checked. These three sensors get real time information from the commercial telecare sensors installed at UPM Accessible Smart Home and supplied by the manufacturer (Tunstall). The middle box gets a record of the Ambient Sensors (Estado de los Sensores Ambientales) and shows a checked open tap sensor (Sensor grifos abiertos), placed in the Bathroom.

For this use case, the system took 1.2 s to propose (Propuestas box, upper right) bathroom temperature regulation to prevent hypothermia and closing taps as internal tasks. It also triggers a call to the SOS service and gives probabilities (Probabilidades Turambar at the lower right) of 0.8723, 0.4002 and 0.1490, respectively, to decide a severe risk situation, send a message to a relative and call the firemen.

The uncertainty engine does not suggest calling the firemen (probability 1 instead of 0.1490) as the Flood Sensor (Sensor inundación) is not checked in the example of Figure 10. Once the water level reaches over 1 mm, the Tunstall sensor validated at the SH activated a signal and TALISMAN on the floor + hybrid reasoner proposed to call the firemen and increased the probability of a severe risk situation.

The goodness of the system outputs were discussed with a psychologist in chief, two social workers and two home telecare experts who work at the Spanish Red Cross Society, which is an entity that has operated public home Telecare services in Spain since the mid-nineties. Use cases were reviewed and discussed in an iterative way during three months to ensure proper outputs to the 128 combinations of personal sensors and 2048 different states of ambient sensors placed in the UPM Accessible Smart Home.

## 4. Conclusions

This research provides a hybrid reasoning system for AAL in the Smart Home based on context aware models to support personal autonomy and promote independent living. The deployed framework and integrated solution manages heterogeneous sensor inputs, deductions and actions to be triggered by the intelligence engine. A user-oriented approach helps to detect events detected by biomedical devices and environmental sensors and make decisions according to the SH context. The use of ontology-based reasoners allows us to represent the knowledge base of the context aware system that extracts relevant parameters to monitor the person at home in a private way. TALISMAN+ permits processing and checking of environment, personal and physiological data collected in real time through low-cost devices and allows the early detection of risk situations and the notification of external caregivers.

A combination of 128 and 2048 sets of input variables led us to test multiple use cases that represent heterogeneous context situations and request specific outputs. The evaluation process was carried out along three months to analyze and compare the expected behavior according to the criteria of five different experts in home telecare and independent living. Even though the system displayed a reliable behavior, the obtained results indicated the existence of some cases in which the information is not accurate enough to understand the situation in the house. In these cases, other techniques to obtain appropriate deductions in less accurate contexts, such as fuzzy logic, are being integrated. Scalable integration of TALISMAN+ at the UPM Accessible Digital Home and UA MetalTIC house allowed the study of sustainable homecare services with users from the Parkinson's and Spinal Cord Injury associations. Implemented local and remote ontologies facilitate data reasoning and interoperability with the SH monitoring systems.
